# Molecular surveillance of respiratory viruses with bioaerosol sampling in an airport

**DOI:** 10.1186/s40794-018-0071-7

**Published:** 2018-09-17

**Authors:** Emily S. Bailey, Jessica Y. Choi, Juliana Zemke, Myagmarsukh Yondon, Gregory C. Gray

**Affiliations:** 1 Duke Global Health Institute, Duke University, 310 Trent Drive, Durham, North Carolina 27710 USA; 2 Division of Infectious Diseases, Duke University School of Medicine, DUMC Box 102359, Durham, North Carolina 27710 USA; 3 Global Health Research Center, Duke-Kunshan University, No. 8 Duke Avenue, Kunshan, Jiangsu China; 4 Emerging Infectious Diseases Program, Duke-NUS Medical School, 8 College Road, Singapore, 169857 Singapore

**Keywords:** Respiratory viruses, Bioaerosol, Epidemiology, Air travel

## Abstract

Recognizing that crowded, high-traffic airports and airplanes have been implicated in respiratory disease transmission, we partnered with administrators of Raleigh Durham International Airport (RDU) in conducting a pilot study of aerosol surveillance for respiratory viruses at RDU. From January to March 2018 we used NIOSH 2-stage samplers to collect 150 min aerosol samples in crowded areas at RDU. Four (17%) of the 24 samples were positive for known respiratory pathogens including influenza D virus and adenovirus. These results suggest the feasibility of employing bioaerosol surveillance techniques in public transportation areas, such as airports, as a noninvasive way to detect and characterize novel respiratory viruses.

**Electronic supplementary material:**

The online version of this article (10.1186/s40794-018-0071-7) contains supplementary material, which is available to authorized users.

## Introduction

As the number of individuals who travel by air increases, issues regarding air quality and the potential risk of respiratory infection during travel and flight have become increasingly important. In the last 20 years alone, there have been outbreaks of several important respiratory viruses that have had a major effect on air travel. A few examples include influenza A(H1N1) pdm09 virus, severe acute respiratory syndrome coronavirus (SARS-CoV), middle east respiratory syndrome coronavirus (MERS-CoV), and emerging H7N9 avian influenza A viruses. The outbreak of SARS coronavirus for example can be tracked from the Hotel Metropole in Hong Kong to areas where infected guests traveled by air after staying at the hotel [1]. This risk of the spread of pathogens by air travel has influenced the development of airport passenger screening technologies at airports. Many airports use thermal scanning to screen incoming passengers for febrile illness. In general, these techniques are not entirely effective in preventing the spread of viral infectious disease because passengers, either with a fever or presenting symptoms with respiratory virus infections such as influenza virus, often shed viruses before they have fever. A notable example of the failure of screening methods occurred in 2009 during the spread of influenza A(H1N1) pdm09 virus, where passengers on a flight from Cancun Mexico were screened but still managed to infect other passengers during flight [2].

In response to the concern surrounding the spread of infectious disease through air travel, a 2017 National Academy of Science report has highlighted the need for additional research and evaluation of the exit screening procedures designed to protect public health from disease threats [3]. The United States in particular has developed a biomonitoring program that detects pathogens in air samples; however, this system (the US Biowatch) is known to be error prone with numerous false positives frequently detected [4]. An alternative response to this need for public health screening is to concentrate and examine air samples, using personal or standalone air samplers. This bioaerosol sampling technique has been adapted for respiratory virus screening in swine production facilities [5], poultry markets [6], clinical settings [7], and airports [8]. In this pilot study, we studied bioaerosol samples collected in Raleigh Durham International Airport for molecular evidence of respiratory viruses. This international airport sees approximately 11.6 million passengers per year, or about 32,000 per day and uses a high throughput exhaust system (including high efficiency particulate air, HEPA, filters) [9]. Our overall goal was to determine if environmental air sampling is a viable method for respiratory virus detection in airport settings.

## Methods and materials

### Ethics approval and study location

“Exemption from review” status was granted to this study by the Institutional Review Board at Duke University, given that the methods involved did not include contact with human subjects. Permission and collaboration from the appropriate coordinating bodies at Raleigh-Durham International Airport (RDU) was sought and obtained prior to the start of the collection period. Staff members at RDU assisted in the coordination of sampling devices during the study period.

### Bioaerosol sampling

Sampling at RDU Terminal 2 took place during a 9-week period from January 10th to March 7th, 2018. During this time, 12 sampling sessions of 150 min resulted in 24 sample collections generating 72 individual samples to be analyzed for a panel of respiratory viruses.

The sampling devices were NIOSH BC 251 Personal Aerosol Samplers that featured two stages of collection and a polytetrafluoroethylene (PTFE) back-up filter (.03 μm pore, 37 mm). The two stages filtered pathogens greater than 4 μm or 1–4 μm in size and the back-up filter captured pathogens less than 1 μm in size. Thus, each sampler produced three samples per sampling period. An AirCheck XR5000 Sample Pump (Cat. # 210–5000, SKC, Inc., Eighty-Four, PA) was used to circulate environmental air through the samplers at a calibrated rate of 3.5 l per minute throughout the sampling period.

The sampling periods were scheduled during high-traffic times of domestic and international flight arrivals and departures. The samplers were set up on tripods approximately 1.5 m above the ground (approximately breathing level), at two different locations within the RDU Baggage Claim areas (between baggage carousels that were the focal points of the arriving flyers) and at each end of the terminal departure gates. During each sampling period, environmental temperature, humidity, and general meteorological outlook were noted. At the end of each session, the location, time, sampler number, pump number, and run time was recorded for each sampling device.

### Sampling processing

Samples were transported back to the Duke One Health Research Laboratory immediately following sample collection. At this time, sterile virus collection medium (PBS with 0.5% BSA) was used to soak the filters and catchment containers for each stage. This medium was then aliquoted into 2.0 mL cryovials, which were promptly stored at − 80 °C for future molecular analyses.

### Molecular assays

Detection of influenza A/B/C/D, human enterovirus, human adenovirus, and human coronavirus was performed using previously published real-time polymerase chain reaction (qPCR) and real-time reverse transcription polymerase chain reaction (qRT-PCR) assays with DNA or cDNA positive controls and negative controls of nuclease-free water (Additional file 1: Table S1).

Viral RNA was extracted from stored samples with the QIAmp Viral RNA Mini Kit (QIAGEN, INC., Valencia, CA), and then analyzed with qRT-PCR assays utilizing Superscript® III Platinum One-Step qRT-PCR System with Platinum® Taq DNA Polymerase (Thermo Fisher Scientific, Inc., Waltham, MA) for influenza A [10, 11], influenza B [10, 12], influenza C [13], influenza D [10], human coronavirus [14, 15], and human enterovirus [14]. Gel-based RT-PCR assays detected for pan-species coronaviruses [14] with Superscript® III Platinum One-Step RT-PCR System with Platinum® Taq DNA Polymerase (Thermo Fisher Scientific, Inc., Waltham, MA).

Viral DNA was extracted utilizing the QIAamp DNA Blood Mini Kit (QIAGEN, Inc. Valencia, CA), then analyzed for human adenovirus [15] by real-time PCR (qPCR) assay [16] with the Sso Advanced Universal Probes Supermix (Bio-Rad, Hercules, CA). Adenovirus-positive specimens were further confirmed using previously described two-step molecular assays with focus on the hexon gene [17]. Analysis for pan-species adenovirus [18] was performed on extracted viral DNA using the Platinum® Taq DNA Polymerase Kit (Thermo Fisher Scientific Inc., Waltham, MA). Sequencing of resultant amplified product from both the pan-species assay and the hexon assay was performed by Eton Bioscience (Eton Bioscience, Inc., Raleigh, NC, USA). Sequences were then aligned, edited, and compared to the NCBI sequence database using the BLAST application of BioEdit 7.1.9 (Ibis Biosciences, Carlsband, CA, USA).

## Results

A total of 24 NIOSH 2-stage samples were collected (Table 1). Overall, 4 (17%) out of 24 samples indicated evidence of at least 1 respiratory pathogen, with 1 (4.1%) positive for influenza D and 3 (12.5%) positive for adenovirus. Samples were most frequently positive in the 4 μm or 1–4 μm particle size ranges with one adenovirus and the influenza D sample detected at 4 μm and two adenovirus positives detected at 1–4 μm. Two (67%) of the 3 adenovirus-positive specimens, samples 16 and 19, were successfully sequenced using the panadenovirus assay and were found to be human adenovirus type 1 (NCBI accession number AF534906.1). Adenoviruses were detected more frequently in the baggage claim 2 area, with 2 (2.8%) positive samples detected in that area. None of the 72 samples were positive for influenza A, influenza B, influenza C, or coronaviruses. Although not the focus of our study, we did not detect viable viruses using culture analysis associated with positive aerosol samples at RDU airport.

**Table 1:** Molecular results for aerosol sampling four sites in RDU Airport, January–March, 2018

Sample ID	Temp (°F)	RH (%)	Site	FluA	FluB	FluC	FluD	AdV	PanAdV	CoV	PanCoV
1	74.0	32.1	Baggage Claim 1	–	–	–	–	–	–	–	–
2			Baggage Claim 2	–	–	–	–	–	–	–	–
3	68.9	44.5	Baggage Claim 1	–	–	–	–	–	–	–	–
4			Baggage Claim 2	–	–	–	–	–	–	–	–
5	72.1	26.8	Terminal 2A	–	–	–	–	–	–	–	–
6			Terminal 2B	–	–	–	–	–	–	–	–
7	63.6	25.1	Terminal 2A	–	–	–	–	–	–	–	–
8			Terminal 2B	–	–	–	–	–	–	–	–
9	71.0	38.0	Baggage Claim 1	–	–	–	–	–	–	–	–
10			Baggage Claim 2	–	–	–	–	–	–	–	–
11	73.4	52.2	Terminal 2A	–	–	–	–	–	–	–	–
12			Terminal 2B	–	–	–	–	–	–	–	–
13	ND	ND	Baggage Claim 1	–	–	–	+	–	–	–	–
14			Baggage Claim 2	–	–	–	–	–	–	–	–
15	70.07	39.5	Terminal 2A	–	–	–	–	–	+	–	–
16			Terminal 2B	–	–	–	–	–	+	–	–
17	71.4	60.4	Terminal 2A	–	–	–	–	–	–	–	–
18			Terminal 2B	–	–	–	–	–	–	–	–
19	78.6	52.9	Baggage Claim 1	–	–	–	–	–	+	–	–
20			Baggage Claim 2	–	–	–	–	–	–	–	–
21	77.1	36.0	Baggage Claim 1	–	–	–	–	–	–	–	–
22			Baggage Claim 2	–	–	–	–	–	–	–	–
23	71.2	30.8	Baggage Claim 1	–	–	–	–	–	–	–	–
24			Baggage Claim 2	–	–	–	–	–	–	–	–

Abbreviations: *Temp* temperature, *ND* not detected, *RH* relative humidity, *FluA* influenza A virus, *FluB* influenza B virus, *FluC* influenza C virus, *FluD* influenza D virus, *AdV* adenovirus, *CoV* coronavirus

During sampling, air temperature ranged from 63.6 °F to 78.6 °F and the relative humidity (RH) ranged from 25.1 to 60.4% (Table 1). There was no statistically significant difference between the mean air temperature or the mean RHs between the groups of samples that were positive or negative for respiratory viruses.

## Discussion

In this pilot aerosol study, we conducted surveillance for human and zoonotic respiratory viruses in an airport setting over a period of nine weeks from January to March 2018. We detected respiratory viruses in 17% of the aerosol samples collected. Through our surveillance, we observed molecular evidence of influenza D virus and adenovirus in aerosol samples through non-invasive and non-disruptive environmental sampling techniques. This sampling approach was particularly important in an airport setting, where large numbers of passengers either sat near or passed by the samplers in the terminal or baggage claim.

This study of aerosols conducted in an airport is likely the first of its kind in the United States. Our detection of adenoviruses in aerosol samples is comparable to similar studies conducted in an airport and other settings [5, 7]. Although respiratory adenoviruses are not typically considered to have as much of an impact as other respiratory viruses, such as influenza viruses, well-documented outbreaks of novel adenovirus strains causing severe respiratory infections have occurred [19]. Despite the fact that the adenoviruses detected in this study were detected using our panspecies molecular assay, upon sequencing, they were identified as human adenoviruses. We did not find evidence of novel or zoonotic viruses in aerosol samples, potentially due to the high throughput ventilation system in place at RDU airport. Another limitation of this study was the inability to link aerosol results with individual passengers, or individual planes. Using this detection method, we were only able to detect viruses from one source (aerosols). As the airport is a high traffic area, and the focus of this study was focused on environmental air sampling, it is not clear which people were harboring the detected viruses. Additionally, as this airport uses a high throughput (HEPA filtered) ventilation system it was not possible to determine the effect of high quality air circulation on our virus detection based on the methods described here and we may not have captured all virus present at the sampling locations.

Despite these limitations, the results of this study suggest that aerosol sampling is a useful technique for respiratory virus surveillance in high traffic and crowded areas such as airports. Aerosol sampling has advantages in that it minimally interrupts walking traffic in busy places, is simple in setup and operation, and the processing procedures for isolating the viral nucleic acid is relatively simple [7]. Together with our finding that 17% of collected aerosol samples showed molecular evidence for at least one respiratory pathogen and the ease of aerosol sampler use, our findings suggest that travelers may be sharing respiratory viruses in airports and other high-traffic areas. Such areas may, therefore, benefit from aerosol sampling to strengthen surveillance to protect the public from respiratory viruses. This strengthened surveillance may also support public health responses to respiratory virus detection by allowing airports, or other high traffic areas employing these methods, to recommend interventions (such as respirators or masks) to at risk travelers.

## Additional file

Additional file 1: **Table S1.** Primer and probe sequences for rPCR and rRT-PCR. Full primer and probe sequences for rPCr and rRT-PCR, including references. (DOCX 13 kb)

## Abbreviations

(BLAST): Basic Local Alignment Search Tool
(cDNA): complementary deoxyribonucleic acid
(DNA): deoxyribonucleic acid
(MERS-CoV): middle east respiratory syndrome coronavirus
(NCBI): National Center for Biotechnology Information
(NIOSH): National Institute of Occupational Safety and Health
(PTFE): polytetrafluoroethylene
(qPCR): real-time polymerase chain reaction
(qRT-PCR): real-time reverse transcription polymerase chain reaction
(RDU): Raleigh Durham International Airport
(RH): Relative humidity
(SARS-CoV): severe acute respiratory syndrome coronavirus
(US): United States
