# Supplementary material for: Molecular surveillance of respiratory viruses with bioaerosol sampling in an airport
Source: Trop Dis Travel Med Vaccines. 2018 Sep 17;4:11. doi: 10.1186/s40794-018-0071-7 (PMC6142699; doi:10.1186/s40794-018-0071-7)
Supplement: Supplementary file 1 — Table S1. Primer and probe sequences for rPCR and rRT-PCR. Full primer and probe sequences for rPCr and rRT-PCR, including references. (DOCX 13 kb) [file 40794_2018_71_MOESM1_ESM.docx]

| **Table S1: Primer and probe sequences for rPCR and rRT-PCR.** | | | |  |  |
| --- | --- | --- | --- | --- | --- |
|  | **Forward primer(s)** | **Reverse primer(s)** | **Probe(s)** | **Gene or Target** | **Reference** |
| **IAV** | 5’-GAC-CRA-TCC-TGT-CAC-CTC-TGA-C-3’ | 5’-AGG-GCA-TTY-TGG-ACA-AAK-CGT-CTA-3’ | 5’-FAM-TGC-AGT-CCT-CGC-TCA-CTG-GGC-ACG-BHQ 1-3’ | Matrix | Centers for Diseases Control and Prevention, 2017 |
| **IBV** | 5’-TCC-TCA-AYT-CAC-TCT-TCG-AGC-G-3’ | 5’-CGG-TGC-TCT-TGA-CCA-AAT-TGG-3’ | 5’-FAM-CCA-ATT-CGA-GCA-GCT-GAA-ACT-GCG-GTG-BHQ 1-3’ | Matrix | Centers for Disease Control and Prevention, 2017 |
| **ICV** | 5’-TGGGAGAGATGGTGTGGAGATA-3’ | 5’-TCTTTTTCCATCGAGTCAATTTCA-3’ | 5’-FAM-AAAGACCACAATTATGC-IBFQ-3’ | Matrix | Hause *et al*., 2013 |
| **IDV** | 5’-GCTGTTTGCAAGTTGATGGG-3’ | 5’-TGAAAGCAGGTAACTCCAAGG-3’ | 5’-FAM-TTCAGGCAAGCACCCGTAGGATT-IBFQ-3’ | C/OK | Pabbaraju *et al*., 2013 |
| **Human**  **CoV** | 5’-GTTCTGATAAGGCACCATATAGG-3’  5’-CATACTCTGACGGTCACAATAATA-3’  5’-TCCTACTAYTCAAGAAGCTATCC-3  5’-CATACTATCAACCCATTCAACAAG-3’ | 5’-TTTAGGAGGCAAATCAACACG-3’  5’-ACCTTAGCAACAGTCATATAAGC-3’  5’-AATGAACGATTATTGGGTCCAC-3’  5’-CACGGCAACTGTCATGTATT-3’ | 5’-TXR-CGCATACGCCAACGCTCTTGAACA-3’  5’-YAK-TGCCCAAGAATAGCCAGTACCTAGT-3’  5’-CY5-TYCGCCTGGTACGATTTTGCCTCA-3’  5’-FAM-ATGAACCTGAACACCTGAAGCCAATCTATG-3’ | NL63, OC43, HKU1, 229E | Loens *et al*., 2012 |
| **HumanEV** | 5’-GGCCCCTGAATGCGGCTAATCC-3’ | 5’-GCGATTGTCACCATWAGCAGYCA-3’ | 5’-FAM-CCGACTACTTTGGGWGTCCGTGT-IBFQ-3’ | 5’NTR | Oberste *et al.*, 2012 |
| **ADV** | 5’-CAG-GAC-GCY-TCG-GAG-TAC-CTG-A-3’ | 5’-CGG-TGG-TCA-CAT-CGT-GGG-T-3’  5’-GCT-GAA-GTA-CGT-VTC-GGT-GGC-3’  5’-GGT-GAA-GTA-GGT-GTC-CGT-GGC-3’ | 5’-FAM-TGG-TGC-AGT-TYG-CCC-G-MGB(NFQ)-3’ | Hexon | Bil-Lula *et al.*, 2012 |
| adenovirus (ADV); coronavirus (CoV); enterovirus (EV); influenza A virus (IAV); influenza B virus (IBV); influenza C virus (ICV); influenza D virus (IDV). | | | | | |
